# Clinical predictors of multiple failure to biological therapy in patients with rheumatoid arthritis

**DOI:** 10.1186/s13075-020-02354-1

**Published:** 2020-12-09

**Authors:** Marta Novella-Navarro, Chamaida Plasencia, Carolina Tornero, Victoria Navarro-Compán, José L. Cabrera-Alarcón, Diana Peiteado-López, Laura Nuño, Irene Monjo-Henry, Karen Franco-Gómez, Alejandro Villalba, Alejandro Balsa

**Affiliations:** 1 Rheumatology Department, Hospital Universitario La Paz, Paseo de la Castellana 261, 28046 Madrid, Spain; 2 Bioinformatic Unit (GENOXPHOS-group) Centro Nacional de Investigaciones Cardiovasculares (CNIC), Madrid, Spain

**Keywords:** Rheumatoid arthritis, Biological therapy, Refractory disease

## Abstract

**Background:**

Biological therapies have improved the clinical course and quality of life of rheumatoid arthritis (RA) patients. Despite the availability and effectiveness of these treatments, some patients experience multiple failures to biologic disease-modifying antirheumatic drugs (bDMARDs), constituting a particular challenge to clinicians.

**Objectives:**

This study aims to determine the percentage of rheumatoid arthritis (RA) patients who fail to respond to subsequent bDMARDs, describe their characteristics, and identify specific baseline and early features during the first bDMARD as possible predictors of consecutive multiple bDMARD failure.

**Methods:**

This is a longitudinal study involving RA patients from the prospective biological cohort drawn from the La Paz University Hospital RA Registry (RA-Paz), starting a bDMARD during the years 2000 to 2019. Patients who presented insufficient response (due to primary or secondary inefficacy) to at least three bDMARDs or two bDMARDs with different mechanism of action were considered multi-refractory (*MR-patients*). Patients who achieved low disease activity or remission (by DAS-28) with the first bDMARD and maintained this over a follow-up period of at least 5 years were considered non-refractory (*NR-patients*)*.*

**Results:**

A total of 41 out of 402 (10%) patients were MR-patients and 71 (18%) NR-patients. In the multivariate analysis, the presence of erosions, younger age, higher baseline DAS-28 and mostly achieving delta-DAS < 1.2 after 6 months of the first bDMARD (OR 11.12; 95% CI 3.34–26.82) were independently associated with being MR-patients to bDMARDs.

**Conclusions:**

In our cohort, 10% of patients with RA were observed to have multi-refractoriness to bDMARDs. This study supports the contention that younger patients with erosive disease and especially the early absence of clinical response to the first bDMARDs are predictors of multi-refractoriness to consecutive biologics. Hence, patients with these characteristics should be monitored more closely and may benefit from personalized treatments.

## Background

Biologic disease-modifying antirheumatic drugs (bDMARDs) have demonstrated their effectiveness in the treatment of rheumatoid arthritis (RA), improving the clinical course of the disease and patients’ quality of life. The repertoire of bDMARDs is continually expanding, and hence, patients who fail to respond to one bDMARD may change through another treatment in accordance with guidelines that endorse cycling or switching strategies after failure to first bDMARD [1, 2].

Nowadays, despite the increasing number of therapeutic tools available, the successful treatment of certain RA patients remains particularly challenging for clinicians. This is in part due to the severity of the disease, but additionally may be related to the tendency of rheumatologists to cycle or switch therapies in an attempt to achieve strict disease control, following the treat-to-target recommendations [3]. The percentage of patients who fail to respond to multiple bDMARDs in clinical practice is sufficiently relevant to warrant special focus due to the intrinsic difficulties of achieving good disease control. Notably, approximately two thirds of patients fail to respond in the first 6 months of treatment with first tumor necrosis factor inhibitor (TFNi) [4], and at least 12% of patients starting a second bDMARD discontinue treatment due to inefficacy [5].

Numerous studies have tried to establish clinical, serological, and imaging features that could help better define the prognosis of RA [6–9]. These studies have focused on the identification of predictors of response to biologic therapies in terms of achieving remission or low disease activity [6–9]. Nonetheless, few studies have evaluated which characteristics could help identify those RA patients most susceptible to suffering multiple failures to bDMARDs [10, 11].

The aim of the present study was to determine the percentage of patients who fail to respond to multiple biological treatments in our cohort of patients with RA, and to identify their characteristics and specific features at baseline and early stages of the first bDMARD as possible predictors of consecutive multiple failures to bDMARDs.

## Patients and methods

This study involved subjects with RA from the prospective biological cohort of patients drawn from the Rheumatoid Arthritis Registry at La Paz University Hospital between January 2000 and August 2019. Ethical approval was obtained from the La Paz Ethics Committee (PI-1155).

The “Rheumatoid Arthritis La Paz University Hospital” (RA-Paz) Registry is a database of all patients who have received, or who are receiving, treatment with bDMARDs [12]. This database enables rheumatologists to include clinical information on RA patients since the beginning of the bDMARD treatment and during follow-up, monitoring clinical response and adverse events every 6 months.

Inclusion criteria were as follows: age ≥ 18 years RA patients according to the 1987 ACR or 2010 ACR/EULAR classification criteria [13, 14] and treated with any bDMARDs (TNFi, Abatacept, Tocilizumab, and Rituximab).

### Definition for multi-refractory RA patients (MR-patients) and non-refractory patients (NR-patients)

Since there is no consensus on the definition of multi-bDMARD refractoriness, we classified as MR-patients those who presented insufficient response to at least three bDMARDs or two bDMARDs with different mechanism of action. Patients who discontinued bDMARDs due to adverse events, contraindications, or intolerance were considered refractory if disease activity was moderate or high at treatment withdrawal.

In contrast, NR-patients were defined as those who achieved low disease activity or remission with the first bDMARD and continued with the same treatment for at least 5 years. This cut-off was established because the mean time under biological treatment until the development of secondary inefficacy was approximately 4 years in our population. Therefore, although it is a strict criterion, we chose 5 years to ensure that NR-patients were really the group of good responders to the first bDMARD.

Patients who discontinued treatment due to adverse events such as infusional or cutaneous reactions related to drug administration, loss of follow-up, pregnancy, sustained remission (disease remission at least 6 months and 2 consecutive visits), severe infections (infections that require hospital admission), neoplasms (malignancies diagnosed during treatment with bDMARDs), and death were excluded. Likewise, all patients who did not meet the MR or NR criteria, which means patients who failed only one bDMARD or two (with the same mechanism of action) and patients with less than 5 years of follow-up were also excluded from the final analysis.

### Data collection

For all patients, the following data were collected just prior to starting the first bDMARD: demographic characteristics (age, sex, race, body mass index, educational level, smoking habit), comorbidities (diabetes mellitus, dyslipidemia, hypertension, heart disease, chronic lung disease and chronic kidney disease), age at diagnosis of RA, age at starting bDMARDs, previous and concomitant treatments (corticosteroids and conventional synthetic—csDMARDs), presence of bone erosions (assessed by simple radiography), extra-articular manifestations (rheumatoid nodules, pulmonary involvement, ocular manifestations, sicca syndrome, vasculitis), and laboratory parameters such as rheumatoid factor (RF) and anti-citrullinated peptide antibodies (ACPA). In addition, health Assessment Questionnaire (HAQ), C-reactive protein (CRP), Erythrocyte Sedimentation Rate (ESR), tender joint count (TJC), swollen joint count (SJC), and DAS-28-ESR were assessed at baseline and 6 months after starting the first bDMARD. Delta-DAS-28 was also calculated for this period for both MR and NR-patients. Treatment details such as global time on bDMARD(s) for both groups, time on each treatment and reasons for discontinuation in MR-patients were also recorded.

### Statistical analysis

Descriptive analyses of all patients were performed. Using the “multi-refractory status” as the dependent variable, multiple bivariate logistic regression models were performed to identify which features were associated with this outcome and therefore should be considered in the multivariate analyses. Prior to adjusting the multivariate analysis, we performed a multi-collinearity test (Supplementary Table S1) analyzing variance inflation factor (VIF > 1) in order to check for any possible correlations between the independent variables. Those variables significantly associated with the outcome (*p* value < 0.1) and uncorrelated independent variables were assembled into a multivariable logistic regression analysis and finally, through a stepwise strategy, we selected the more accurate multivariate model using the method of maximum likelihood. *p* values < 0.05 were considered statistically significant. Odds ratio (OR) and confidence intervals were also calculated (IBM SPSS 21.0).

## Results

In total, 595 RA patients treated with bDMARDs through the RA-Paz Registry were identified, of which 193 discontinued treatment due to: sustained remission (13.4%), loss of follow-up (9.8%), severe infections (5.1%), neoplasms (8.8%), death (3.1%), pregnancy (3.5%), adverse events including infusional reactions (12.3%), loss of therapeutic adherence, and others (29.1%) (Supplementary Figure S1)*.* Of the 402 patients who remained under treatment at the time of data collection, 290 were excluded because they did not fulfill the selection criteria according to the pre-established definition of multi and non-refractory patients. Finally, 112 patients (41 MR and 71 NR) were included in our analysis.

### Demographic and clinical characteristics at the start of first bDMARD: MR-patients vs NR-patients

Patient characteristics at baseline, prior to start a first bDMARD, are shown in Table 1. No differences in age, gender, or age at diagnosis were found between the two groups. In MR-patients, shorter disease duration between RA diagnosis and starting bDMARD (6.9 vs 10.0 years, *p* = 0.04) and a higher number of previous cDMARDs were observed. Erosions and extra-articular manifestations were more frequent in MR-patients (58.5% vs 25.4%, *p* = 0.03 and 29.3% vs 12.7%, *p* < 0.001 respectively). Differences between disease activity at baseline were also observed, MR-patients presented higher DAS-28 (5.8 ± 1.2 vs 5.1 ± 1.0, *p* = 0.002).

**Table 1:** Demographic and clinical characteristics of patients at the start of first bDMARD in all patients included in the analysis and separately for multi-refractory patients and non-refractory patients

	Total***, n*** = 112	Multi-refractory patients (MR-patients)***, n*** = 41	Non-refractory patients (NR-patients)***, n*** = 71	***p*** value
**Sex (female)**	95 (84.8)	33 (80.5)	62 (87.3)	0.33
**Smoking status**				
**Never smoker**	63 (56.3)	22 (53.7)	41 (57.7)	
**Smoker**				
**Past**	23 (20.5)	12 (29.3)	11 (15.5)	0.17
**Current**	26 (23.2)	7 (17.0)	19 (26.8)	
**BMI (kg/m**^**2**^**)**	26.4 (5.1)	26.6 (5.9)	26.2 (4.6)	0.79
**Age (years)**				
**At diagnosis**	43.7 (13.1)	43.0 (13.6)	44.2 (12.8)	0.63
**At starting cDMARD**	46.4 (12.8)	45.1 (13.5)	47.2 (12.4)	0.41
**At starting bDMARD**	52.6 (11.9)	49.9 (12.3)	54.1 (11.5)	0.07
**Extra-articular manifestations**	21 (18.8)	12 (29.3)	9 (12.7)	0.03*
**Immunological parameters**				
**Positive RF (IU/ml)**	99 (88.4)	35 (85.4)	64 (90.1)	0.44
**Positive ACPA (IU/ml)**	94 (83.9)	33 (80.5)	61 (85.9)	0.45
**Erosions**	42 (37.5)	24 (58.5)	18 (25.4)	0.001*
**Concomitant cDMARD**	89 (79.5)	35 (85.4)	54 (76.1)	0.24
**Number of previous cDMARDs**				
**< 3**	75 (66.9)	16 (39.2)	59 (83.1)	
**≥ 3**	37 (33.1)	25 (60.8)	12 (16.9)	0.001*
**Disease duration between diagnosis and bDMARD (years)**	8.9 (7.7)	6.9 (6.8)	10.0 (8.1)	0.04*
**Concomitant steroids**	112 (100)	41 (100)	71 (100)	–
**First bDMARD**				
**TNFi**	95 (84.8)	38 (92.7)	57 (80.3)	0.07
**Non-TNFi**	17 (15.2)	3 (7.3)	14 (19.7)	
**Prior to start 1st bDMARD**				
**DAS-28**	5.4 (1.1)	5.8 (1.2)	5.1 (1.0)	0.002*
**Tender joint count**	9.9 (7.1)	12.3 (7.7)	8.5 (6.2)	0.005*
**Swollen joint count**	8.3 (4.8)	9.8 (6.2)	7.4 (3.5)	0.02*
**HAQ**	9.9 (5.2)	11.9 (5.6)	5.5 (5.1)	0.003*
**ESR (mm/h)**	33.1 (20.2)	37.1 (21.4)	30.8 (19.3)	0.11
**CRP (mg/dl)**	12.4 (16.8)	16.7 (23.0)	10.1 (11.6)	0.05*

* statistically significant

Results are shown as mean (standard deviation) for continuous variables and absolute number (percentage) for categorical variables

*BMI* body mass index, *RF* rheumatoid factor, *ACPA* anti-citrullinated peptide antibodies, *cDMARD* conventional disease-modifying antirheumatic drug, *bDMARD* biological disease-modifying antirheumatic drug, *DAS-28* Disease Activity Score-28, *HAQ* Health Assessment Questionnaire, *ESR* erythrocyte sedimentation rate, *CPR* C-reactive protein

### Clinical response to biological therapy: MR-patients vs NR-patients

The percentage of subjects who responded during first 6-months of the initial treatment was lower in MR-patients than in NR-patients (43.9% vs 77.5%, *p* = 0.001). Global time on bDMARDs treatment was longer in MR-patients than NR-patients (11.7 vs 9.7 years, *p* = 0.01). For MR-patients, survival time on the first bDMARD measured 4.1 ± 3.4 years, which decreased with the successive treatments up to a mean survival time of 2.1 years with the third biological onwards. The main reason for treatment discontinuation was secondary inefficacy, which was observed in approximately 75% of patients (Supplementary Table S2-S3).

### Risk factors associated with MR

Bivariate analyses showed direct associations between age at the beginning of treatment, time-span since RA diagnosis and initiating bDMARD treatment, presence of erosions and extra-articular manifestations, number of previous cDMARDs, TJC, SJC, DAS-28, and HAQ. Nevertheless, after checking for multi-collinearity, some of these variables were removed due to the correlations between them. For example, a strong correlation between the number of previous cDMARDs and age at first bDMARD was noted. Other observations strongly linked the presence of erosions to the time between diagnosis and bDMARD starting, disease activity, and were associated with HAQ.

Finally, the multivariate analysis showed that being younger (OR 0.95; 95%CI 0.90–0.99), presence of erosions (OR 3.26; 95%CI 1.18–9.00), and higher baseline DAS-28 (OR 2.29; 95% CI 1.39–3.76) prior to starting the first bDMARD and achieving a delta-DAS-28 < 1.2 6 months after starting the first bDMARD (OR 11.12; 95% CI 3.34–26.82) were independently associated with being a MR-patient (Table 2).

**Table 2:** Bivariate and multivariate analysis for the association between demographic and clinical characteristics and multi-refractoriness

VARIABLE	Bivariate, OR (95%CI)	Multivariate, OR (95%CI)
**Age at diagnosis**	0.99 (0.96–1.0)	–
**Sex (Female vs male)**	1.67 (0.58–4.73)	5.94 (0.92–38.20)
**Age at cDMARD treatment**	0.98 (0.95–1.01)	–
**Age at bDMARD treatment**	0.97 (0.93–1.00)	0.95 (0.90–0.99)
**Time between diagnosis and bDMARD treatment**	0.94 (0.89–1.00)	–
**BMI (kg/m**^**2**^**)**	1.01 (0.94–1.09)	–
**Comorbidities**	1.05 (0.71–1.55)	–
**Erosions (ref yes)**	4.07 (1.79–9.26)	3.26 (1.18–9.00)
**Extra-articular manifestations (ref yes)**	2.81 (1.0–7.52)	2.14 (0.59–7.78)
**RF (ref positive)**	0.63 (0.19–2.04)	–
**ACPA (ref positive)**	0.67 (0.24–1.87)	–
**Concurrent metothrexate (ref yes)**	1.83 (0.66–5.10)	–
**Previous cDMARDs**	3.54 (2.05–6.1)	–
**ESR (mm/h)**	1.01 (0.99–1.03)	–
**CRP (mg/dl)**	1.02 (0.99–1.05)	–
**Tender joint count**	1.08 (1.02–1.14)	–
**Swollen count joint**	1.11 (1.02–1.21)	–
**DAS-28 baseline**	1.77 (1.2–2.6)	2.29 (1.39–3.76)
**DAS-28 at 6 month**	2.29 (1.59–3.29)	–
**ΔDAS-28 (ref < 1.2)**	0.22 (0.09–0.52)	11.12 (3.34–26.82)
**HAQ**	1.13 (1.03–1.23)	1.09 (0.92–1.29)
**Smoking status (ref never smokers)**	1.18 (0.54–2.55)	–

*BMI* body mass index, *cDMARD* conventional disease-modifying antirheumatic drug, *bDMARD* biological disease-modifying antirheumatic drug, *RF* rheumatoid factor, *ACPA* anti-citrullinated peptide antibodies, *DAS-28* Disease Activity Score-28, *HAQ* Health Assessment Questionnaire, *ESR* erythrocyte sedimentation rate, *CPR* C-reactive protein

RF positive, > 20 IU/ml; ACPA positive, > 50 IU/ml

## Discussion

Refractory RA constitutes an emerging concern, to date few studies tried to establish the characteristics of RA patients who develop multiple bDMARD failures. Therefore, in the present study, we aimed to provide more data about these patients and we found that being younger at starting bDMARDs, higher baseline DAS-28, presence of erosions and poorer early response within the first 6 months of treatment with bDMARDs (measured by delta-DAS-28) were associated with multi-refractoriness. Nevertheless, it should be noted that our results are based on the definitions we used for MR-patients and NR-patients. According to our definitions, 10% of the patients in the RA-PAZ registry met the condition of multi-refractoriness, compared with 18% of NR-patients. In other cohorts, these percentages vary, and the differences are mainly attributable to heterogeneity in definition of multi-refractory patients, Kearsley-Fleet et al. reported that 6.4% of patients in their study were refractory to multiple DMARDs [10], Bécède et al. found 17% [11], and De Hair et al. estimated 3–10% of difficult-to-treat RA patients in their cohort [15].

Studies attempting to define refractory RA are still scarce, because it is an emerging concept, e.g., Buch considers that multi-refractoriness is due to three different mechanisms: intrinsic refractoriness, pharmacokinetic refractoriness, and false refractoriness in which there is an absence of inflammation, but other biomechanical/degenerative mechanisms are present that influence an insufficient response to treatment [16]. De Hair et al. employed a broader definition of “difficult-to-treat RA” which is considered as a multifactorial condition (immunological, pharmacogenetics, toxicity, comorbidities, etc.) that varies between individuals [15].

Regarding definitions for multi-refractoriness in observational studies, such as ours, these have been based on an arbitrary number of failures to different DMARDs, e.g., Kearsley-Fleet et al. considered patients who initiate their third class of bDMARD to be refractory [10], while Bécède et al. defined as refractory those patients who had an insufficient response to at least 3 DMARDs, and at least one of which was bDMARD [11].

In our study, we wanted to focus on refractoriness to bDMARD and furthermore, we aimed to be more stringent by only considering patients with insufficient response to at least three bDMARD with the same mechanism of action or at least 2 with different target to be refractory. In addition, to avoid confounding factors in the analysis, patients who presented inefficacy to only one bDMARD or two bDMARDs with the same mechanism of action were not classified as multi-refractory. This is also true of those in whom treatment was changed due to adverse events or contraindication, since we wanted to include only those patients with a true inefficacy to treatment. In NR-patients, the follow-up period was 5 years because according to published data, mean bDMARD duration to secondary inefficacy usually takes from 3 to 4 years [17].

Thus, we wanted to ensure that our control group was comprised of a population that met the criteria of sustained response for a long period.

Some studies have shown that high disease activity as measured by the usual indexes (DAS-28, SDAI, CDAI) [18] leads to worse disease prognosis, as well as a poorer response to conventional treatment. However, as previously mentioned, the lack of consensus on the definition of multi-refractoriness makes it difficult for rheumatologist to conduct comparable studies. In our study, disease activity was measured prior to the start of bDMARDs in agreement with other studies that point to a high baseline DAS-28 as a risk factor for the development of bDMARD failure [19]. Nonetheless, we have also found that those patients with a less clinical improvement 6 months after bDMARD treatment (delta-DAS-28 < 1.2) had a higher risk of developing multi-refractoriness. In our cohort, this group, which lacked a significant decrease in DAS-28, had 11-fold greater likelihood of having multiple failures to different bDMARDs. This finding has not been reported in previous studies, although a recent paper that analyzed response trajectories to anti-TNF found that a 6-month response is indicative of future outcomes in RA patients [20].

On the other hand, our results show that established joint damage, as assessed by the presence of bone erosions at the beginning of the biological treatment, was also associated with a poorer response. This makes sense, since such erosions are a feature of more destructive and aggressive joint disease. Indeed, erosions have been shown to be a poor prognostic factor for patients with RA, and hence, they might also be a factor associated with the lack of response to different treatments. In addition, MR-patients received more cDMARDs and bDMARDs were introduced earlier, which reinforce the hypothesis of a more severe disease course. Although treatment with DMARDs is expected to improve disease prognosis, it is difficult to predict which patient with RA will respond adequately to various treatments [1, 21].

Shorter disease duration and being younger prior to start the first bDMARD were also predictors of multi-refractoriness. Previous observational studies in biologic refractory disease [10, 11] also found that shorter disease duration and younger age at bDMARD starting were associated to multi-refractory disease. This is understandable given that these patients present a more aggressive and rapidly progressive disease course. Therefore, they require biological treatment earlier than patients with an adequate response to conventional treatments. On the other hand, this finding may reflect younger patients needing to be treated more aggressively leading to increased switching between bDMARDs in order to stablish a treatment to target that improve outcomes [3, 22].

This study is not without its limitations, mainly because there is no well-established definition of multi-refractoriness [10, 11, 15, 16, 23, 24] and because it is difficult to establish a comparison with other cohorts. Nevertheless, our data can provide more information for the characterization of this subgroup of patients and for definitions of multi-refractory and difficult-to-treat RA which are still pending (although this is one of the objectives of the European League Against Rheumatism (EULAR) task force) [23]. Another limitation could be that factors potentially associated with poorer response to biological therapy (such as chronic pain, fibromyalgia, or depression) were not analyzed separately. The use of composite scores to assess the response to treatment has limitations due to the weight of subjective items in the overall score (DAS-28 in this study), but they are currently the most widely used tool for the assessment of disease activity. Although we did not analyze the component of chronic pain/fibromyalgia in our patients, we observed as baseline characteristics of MR-patients that they had significantly higher levels of CRP and more swollen joints (as objective parameters), although it is true that they also had more tender joints than NR-patients [25–27]. This issue is, however, controversial, since these subjective items can mean that patients are misclassified assuming that they have high disease activity and thus bDMARDs are switched early, potentially accelerating refractoriness.

Furthermore, it would also be interesting to analyze non-multi-refractory patients (i.e., those who have presented a lack of response to a bDMARD) in order to assess whether the baseline clinical characteristics closely resemble MR or NR-patients. Nevertheless, our study’s strengths include its longitudinal design with a long-term follow-up. This allows for the assessment of multi-refractoriness and for the use of strict inclusion criteria in both groups, which confers reliability to the patient characteristics.

In summary, in our cohort, 10% were MR-patients, and certain clinical features such as erosions, being younger at the start of bDMARD treatment, having higher disease activity and, most importantly, poor clinical improvement 6 months after starting the biological treatment may be useful for predicting multiple failures to bDMARDs. That is, we ought to establish possible baseline/early predictors that would allow us to understand and, up to a point, to predict the multi-refractoriness prior to the start of biological treatments. This is because it would be of great interest to clinical practice to avoid inefficient treatments, which entail significant economic burden and potential adverse effects. In addition, this group of patients might benefit from further studies of targeted therapies in order to develop a tailored therapy reducing treatment failure. On the other hand, our findings highlight the fact that a universal standard definition of multi-refractoriness is needed if further studies are going to be capable of establishing predictors to multiple bDMARD failure.

## Supplementary information

**Additional file 1: Supplementary Table S1**. Table performed to check collinearity between significant variables obtained in univariate analysis.

**Additional file 2: Supplementary Figure S1.** Flow-chart of patients included in the study and reasons for discontinuation of treatment. **Supplementary Table S2.** Comparison of clinical activity between refractory and non-refractory patients during treatment with the 1st bDMARD. **Supplementary Table S3.** Time and reasons for discontinuation of bDMARDs in refractory patients.

## Data Availability

The datasets used and/or analyzed during the current study are available from the corresponding author on reasonable request.
