# Supplementary material for: Clinical predictors of multiple failure to biological therapy in patients with rheumatoid arthritis
Source: Arthritis Res Ther. 2020 Dec 9;22:284. doi: 10.1186/s13075-020-02354-1 (PMC7724866; doi:10.1186/s13075-020-02354-1)
Supplement: Supplementary file 1 — Additional file 1: Supplementary Table S1. Table performed to check collinearity between significant variables obtained in univariate analysis. [file 13075_2020_2354_MOESM1_ESM.docx]

| **X**  -------------  **Y** | Age at bDMARD | Time diagnosis-bDMARD | Erosions | Extra-articular  Mfs | Previous cDMARDs | ESR | CRP | Tender joint count | Swollen joint count | Baseline DAS-28 | 6months  DAS-28 | ADAS-28 | HAQ |
| --- | --- | --- | --- | --- | --- | --- | --- | --- | --- | --- | --- | --- | --- |
| Age at bDMARD | - | P=0.06  B=0.26  SE=0.14 | P=0.14  B=3.43  SE=2.32 | P=0.91  B=-0.29  SE=2.9 | **P=0.01**  **B=-2.77**  **SE=1.12** | P=0.35  B=0.05  SE=0.05 | P=0.95  B=0.04  SE=0.07 | P=0.19  B=-0.12  SE=0.16 | P=0.61  B=0.09  SE=0.23 | P=0.34  B=-0.9  SE=1 | P=0.44  B=-0.62  SE=0.78 | P=0.16  B=-3.28  SE=2.36 | P=0.47  B=0.16  SE=0.22 |
| Time diagnosis-bDMARD | P=0.06  B=0.17  SE=0.06 | - | **P<0.001**  **B=5.81**  **SE=1.42** | P=0.76  B=-0.57  SE=1.89 | P=0.83  B=-0.16  SE=0.75 | P=0.67  B=0.05  SE=0.03 | P=0.06  B=-0.87  SE=0.04 | P=0.11  B=-0.15  SE=0.14 | P=0.75  B=-0.16  SE=0.15 | P=0.12  B=-0.98  SE=0.64 | P=0.17  B=-0.70  SE=0.51 | P=0.86  B=0.25  SE=1.55 | P=0.58  B=0.05  SE=0.14 |
| Erosions | P=0.14  B=0.02  SE=0.01 | P=0.14  B=0.02  SE=0.01 | - | P=0.13  B=0.73  SE=0.49 | P=0.09  B=0.33  SE=0.21 | P=0.03  B=0.02  SE=0.01 | P=0.64  B=-0.06  SE=0.01 | P=0.04  B=0.05  SE=0.02 | P=0.01  B=0.16  SE=0.04 | P=0.03  B=0.39  SE=0.18 | P=0.008  B=0.39  SE=0.14 | P=0.06  B=-0.77  SE=0.41 | P=0.04  B=0.08  SE=0.04 |
| Extra-articular mf | P=0.91  B=0.002  SE=0.2 | P=0.75  B=-0.01  SE=0.03 | P=0.13  B=0.73  SE=0.49 | - | P=0.01  B=0.61  SE=0.24 | P=0.11  B=0.01  SE=0.01 | P=0.13  B=-0.03  SE=0.02 | P=0.65  B=-0.16  SE=0.03 | P=0.13  B=0.07  SE=0.04 | P=0.83  B=0.04  SE=0.21 | P=0.04  B=0.34  SE=0.16 | P=0.06  B=-0.91  SE=0.49 | P=0.89  B=0.006  SE=0.04 |
| Previous cDMARD | P=0.01  B=-0.01  SE=0.01 | P=0.83  B=-0.02  SE=0.01 | P=0.09  B=0.32  SE=0.17 | P=0.01  B=0.62  SE=0.23 | - | P=0.56  B=0.03  SE=0.05 | P=0.64  B=-0.04  SE=0.06 | P=0.04  B=0.05  SE=0.01 | P=0.01  B=0.05  SE=0.01 | P=0.02  B=0.28  SE=0.07 | P<0.001  B=0.26  SE=0.06 | P=0.02  B=-0.45  SE=0.19 | P=0.005  B=0.05  SE=0.01 |
| ESR | P=0.35  B=0.12  SE=0.16 | P=0.67  B=0.1  SE=0.24 | **P=0.02**  **B=8.70**  **SE=3.90** | P=0.11  B=7.77  SE=4.44 | P=0.53  B=1.23  SE=1.95 | - | P=0.01  B=0.39  SE=0.14 | P=0.59  B=0.14  SE=0.27 | P=0.1  B=0.65  SE=0.39 | **P<0.001**  **B=8.12**  **SE=1.5** | **P<0.001**  **B=4.72**  **SE=1.27** | P=0.98  B=0.08  SE=0.04 | P=0.02  B=0.91  SE=0.38 |
| CRP | P=0.95  B=0.08  SE=0.13 | P=0.06  B=-0.39  SE=0.21 | P=0.64  B=-1.58  SE=3.44 | P=0.13  B=-6.24  SE=4.15 | P=0.64  B=-0.78  SE=1.71 | P=0.01  B=0.26  SE=0.07 | - | P=0.1  B=0.37  SE=0.23 | P=0.005  B=0.95  SE=0.33 | **P=0.001**  **B=4.71**  **SE=1.36** | P=0.52  B=0.75  SE=1.12 | P=0.21  B=4.33  SE=3.41 | P=0.005  B=0.91  SE=0.32 |
| Tender joint count | P=0.19  B=-0.07  SE=0.05 | P=0.11  B=-0.13  SE=0.08 | **P=0.04**  **B=2.76**  **SE=1.36** | P=0.66  B=-0.77  SE=1.71 | **P=0.004**  **B=1.93**  **SE=0.65** | P=0.49  B=0.01  SE=0.03 | P=0.11  B=0.06  SE=0.04 | - | P<0.001  B=0.81  SE=0.11 | **P<0.001**  **B=5.01**  **SE=0.35** | **P<0.001**  **B=2.29**  **SE=0.43** | P=0.09  B=2.31  SE=1.39 | P<0.001  B=0.67  SE=0.11 |
| Swollen joint count | P=0.61  B=0.01  SE=0.03 | P=0.75  B=-0.18  SE=0.06 | **P<0.001**  **B=3.46**  **SE=0.89** | P=0.1  B=1.87  SE=1.16 | **P=0.01**  **B=1.19**  **SE=0.45** | P=0.16  B=0.03  SE=0.02 | P=0.005  B=0.07  SE=0.02 | P<0.001  B=0.37  SE=0.05 | - | **P<0.002**  **B=2.64**  **SE=0.31** | **P<0.001**  **B=1.23**  **SE=0.29** | P=0.16  B=1.33  SE=0.95 | P=0.02  B=0.28  SE=0.08 |
| Baseline DAS-28 | P=0.34  B=-0.09  SE=0.01 | P=0.13  B=-0.2  SE=0.02 | P=0.02  B=0.48  SE=0.21 | P=0.83  B=0.05  SE=0.27 | P=0.002  B=0.33  SE=0.10 | P<0.001  B=0.02  SE=0.01 | P=0.001  B=0.02  SE=0.01 | P<0.001  B=0.13  SE=0.01 | P<0.001  B=0.14  SE=0.01 | - | P<0.001  B=0.42  SE=0.06 | P=0.03  B=0.47  SE=0.22 | P<0.001  B=0.12  SE=0.01 |
| 6months DAS-28 | P=0.12  B=-0.02  SE=0.01 | P=0.34  B=-0.009  SE=0.001 | P=0.02  B=0.48  SE=0.21 | P=0.83  B=0.05  SE=0.27 | P=0.02  B=0.33  SE=0.11 | P<0.001  B=0.02  SE=0.005 | P<0.001  B=0.02  SE=0.006 | P<0.001  B=0.12  SE=0.09 | P<0.001  B=0.14  SE=0.01 | P<0.001  B=0.42  SE=0.06 | - | P=0.03  B=0.47  SE=0.22 | P<0.001  B=0.12  SE=0.01 |
| ADAS-28 | P=0.16  B=-0.02  SE=0.01 | P=0.86  B=0.004  SE=0.26 | P=0.06  B=-0.77  SE=0.41 | P=0.06  B=-0.91  SE=0.49 | P=0.02  B=-0.46  SE=0.21 | P=0.98  B=0  SE=0.01 | P=0.21  B=0.01  SE=0.01 | P=0.1  B=0.05  SE=0.03 | P=0.16  B=0.06  SE=0.04 | P=0.03  B=0.38  SE=0.18 | P<0.001  B=-0.89  SE=0.19 | - | P=0.98  B=-0.01  SE=0.03 |
| HAQ | P=0.47  B=0.03  SE=0.04 | P=0.58  B=-0.03  SE=0.06 | **P=0.04**  **B=2.11**  **SE=1.04** | P=0.89  B=0.16  SE=1.21 | **P=0.005**  **B=1.45**  **SE=0.51** | P=0.02  B=0.05  SE=0.02 | P=0.005  B=0.08  SE=0.03 | P<0.001  B=0.35  SE=0.06 | P=0.02  B=0.31  SE=0.1 | **P<0.001**  **B=2.46**  **SE=0.37** | **P<0.001**  **B=1.61**  **SE=0.32** | P=0.72  B=-0.38  SE=1.07 | - |

****Supplementary Table S1***. Table performed to check collinearity between significant variables obtained in univariate analysis.
