# Supplementary material for: Clinical predictors of multiple failure to biological therapy in patients with rheumatoid arthritis
Source: Arthritis Res Ther. 2020 Dec 9;22:284. doi: 10.1186/s13075-020-02354-1 (PMC7724866; doi:10.1186/s13075-020-02354-1)
Supplement: Supplementary file 2 — Additional file 2: Supplementary Figure S1. Flow-chart of patients included in the study and reasons for discontinuation of treatment. Supplementary Table S2. Comparison of clinical activity between refractory and non-refractory patients during treatment with the 1st bDMARD. Supplementary Table S3. Time and reasons for discontinuation of bDMARDs in refractory patients. [file 13075_2020_2354_MOESM2_ESM.docx]

***Suplementary Figure S1.*** *Flow-chart of patients included in the study and reasons for discontinuation of treatment.*

Remission 26

Loss of follow-up 19

Inefficacy 13

Death 6

Neoplasms 17

Recurrent infections 10

Adverse events including Infusional reactions 24

Pregnancy 7

Other diseases 17

Other causes 57

402 RA patients under active treatment

*NR: No refractory patients; MR: multi-refractory patients.*

***Supplementary Table S2****. Comparison of clinical activity between refractory and non-refractory patients during treatment with the 1st bDMARD*

|  | Refractory patients  n= 41 | Non refractory patients  n= 71 | p-value |
| --- | --- | --- | --- |
| Prior to 1st bDMARD | | | |
| DAS-28 | 5.8 (1.2) | 5.1 (1.0) | 0.002 |
| HAQ | 11.9 (5.6) | 5.5 (5.1) | 0.003 |
| CRP | 16.7 (23.0) | 10.1 (11.6) | 0.05 |
| 6 months after starting 1st bDMARD | | | |
| DAS-28 | 4.5 (1.5) | 3.1 (1.1) | 0.001 |
| ΔDAS28 | 1.3 (1.4) | 2.1 (1.1) | 0.02 |
| ΔDAS28 >1.2 | 18 (43.9) | 55 (77.5) | 0.001 |
| HAQ | 9.8 (4.8) | 5.5 (5.1) | <0.001 |
| CRP | 7.7 (12.1) | 3.2 (4.7) | 0.03 |

*Results are shown as mean (standard deviation) for continuous variables and absolute number (percentage) for categorical variables.*

*Supplementary table S2. Time and reasons for discontinuation of bDMARDs in*

*refractory patients.*

| 1st bDMARD (n=41) | | |  |
| --- | --- | --- | --- |
| Time on treatment (years) | 4.1 (3.4) |  | |
| Reasons for discontinuation  Primary inefficacy  Secondary inefficacy | 5 (12.2)  36 (87.8) |  | |
| 2nd bDMARD (n=41) | | |  |
| Time on treatment | 2.1 (2.1) |  | |
| Reasons for discontinuation  Primary inefficacy  Secondary inefficacy | 10 (24.4)  31 (75.6) |  | |
| 3rd bDMARD (n=41) | | |  |
| Time on treatment | 2.1 (1.8) |  | |
| Reasons for discontinuation  Primary inefficacy  Secondary inefficacy | 5 (12.2)  20 (48.8) |  | |
| 4th bDMARD (n= 24) | | |  |
| Time on treatment | 3.7 (3.1) |  | |
| Reasons for discontinuation  Primary inefficacy  Secondary inefficacy | 0  6 (5.1) |  | |
| >4th bDMARD (n= 9) | | |  |
| Time on treatment | 2.7 (1.2) |  | |

*Results are shown as mean (standard deviation) for time on treatment and absolute number (percentage) in reasons for discontinuation (primary and secondary inefficacy)*
